# Students with inattention and their experiences of autonomy in learning activities: an interview study with two students and their teachers

**DOI:** 10.3389/fpsyg.2025.1624279

**Published:** 2025-10-24

**Authors:** Marit Uthus, Audhild Løhre

**Affiliations:** ^1^Department of Education and Lifelong Learning, Norwegian University of Science and Technology, Trondheim, Norway; ^2^Department of Teacher Education, Norwegian University of Science and Technology, Trondheim, Norway

**Keywords:** attention deficit hyperactivity disorder (ADHD), inattention, concentration, student autonomy, volition, motivation, autonomy support

## Abstract

**Introduction:**

The purpose of this study was to explore how students diagnosed with attention deficit hyperactivity disorder (ADHD) experience autonomy in learning activities. The study was conducted within the framework of a one-year school-based intervention using an autonomy supportive learning model that included elements of adapted education, self-regulated learning, and teacher autonomy support.

**Method:**

Two students with inattention and two teachers were interviewed about their experiences in the context of the learning model. The interviews were inductively analyzed and self-determination theory proved useful in adding meaning to the students’ experiences.

**Results:**

Analysis showed that in a context that supports their autonomy, the students at times experienced being able to volitionally maintain their concentration. Autonomy support allowed them to address their own needs in terms of interest, enjoyment, and the need for breaks, leading to experiences which can be interpreted as both intrinsic motivation and autonomous extrinsic motivation. Furthermore, ongoing dialogical interactions between students and teachers were highlighted as beneficial to students’ subsequent self-reflections about their needs and what they require to act autonomously in learning activities. However, the expectations of being autonomous learners were at times experienced as a challenge for the students, indicating that autonomy in learning activities that rely on self-regulation might constitute a double-edged sword for students with ADHD. A key contribution of this study lies in its novel application of self-determination theory (SDT) to understand the broader conceptualizations of motivation—particularly internal motives of students with ADHD in mainstream educational settings—responding to recent calls for research that moves beyond deficit-based perspectives. Implications include a need for teachers to increase their competency in differentiated autonomy support. Teachers’ ongoing dialogic interaction with students about their experiences, needs and interests in learning activities should also be a central part of teachers’ competency.

## 1 Introduction

Through decades of research on attention deficit hyperactivity disorder (ADHD), the core symptoms of hyperactivity, inattention, and impulsivity have been recognized to negatively affect students’ motivation and academic outcomes in educational contexts (Tarver et al., 2014; Gray et al., 2017; Arnold et al., 2020; Smith et al., 2023). Academic functioning in school requires a range of skills in areas such as cognition, organization, self-regulation, time-management, and socializing, and the core symptoms of ADHD can lead to impairments in all of these areas (Arnold et al., 2020).

Our focus on autonomy in learning activities aligns with a recent shift within educational psychology in the field of ADHD, which has moved from predominantly addressing negative aspects of the diagnosis and symptom control to also recognizing positive psychological factors and well-being (Newton et al., 2017; Champ et al., 2021; Morsink et al., 2021; Champ et al., 2023). According to self-determination theory (SDT), the satisfaction of the basic psychological need for autonomy is proposed to enhance students’ sense of volition and motivation outcomes in school contexts (see, e.g., Ryan and Deci, 2020), and it is possible that this also applies to students diagnosed with ADHD. A recent study found that students with ADHD symptoms benefit particularly from autonomous engagement (intrinsic motivation) with respect to academic outcome and that interventions that provide these students with autonomy support are especially sought after (Smith et al., 2023). Rogers and Tannock (2018) who recently claimed to be the first to explore perceived autonomy support in a group of students with ADHD symptoms, find that the presence of ADHD symptoms may uniquely interfere with children’s fulfillment of this basic psychological need in the classroom. As this remains an understudied topic, our study contributes to the body of research by exploring how two students with inattention experience autonomy in learning activities, specifically in terms of volitional choice and motivation.

The study was conducted in a Norwegian primary school, within the framework of a one-year school-based intervention using an autonomy supportive learning model that included elements of adapted education and self-regulated learning (SRL). In the Norwegian educational context, inclusive education is high on the agenda with the principle of *tilpasset opplæring* (adapted education) strongly emphasized for all students (Norwegian Ministry of Education and Research, 2024; Norwegian Directorate for Education and Training, 2024). Considering that adapted education seeks to balance individual differentiation with students’ sense of belonging in the community (Norwegian Directorate for Education and Training, 2024), it creates a unique framework for exploring autonomy and autonomy support for students with ADHD in mainstream classrooms. Internationally, however, much of the ADHD literature has focused on behavioral reinforcement and symptom management (Carlson et al., 2002), with limited attention to internal emotions and motivational processes (Morsink et al., 2021; Champ et al., 2023). By applying SDT within the Norwegian framework of adapted education, this study contributes to bridging this gap, offering insights into how autonomy-supportive practices can be tailored to students with ADHD in inclusive settings.

While autonomy is a fundamental psychological need, research suggests that students with ADHD may struggle to benefit from autonomy in learning contexts unless it is accompanied by sufficient structure and support (Rogers and Tannock, 2018). Autonomy, especially when it includes SRL, as in this study, may pose additional challenges for students with ADHD, due to impairments in metacognitive, affective, and behavioral processes (Reddy et al., 2018; Champ et al., 2023).

With the intention of exploring both opportunities and challenges according to autonomy for students with ADHD, the following research question guided our study:

*How do two students with inattention experience autonomy in learning activities, specifically in terms of volitional choice and motivation —and how do their teachers interpret these experiences?*

## 2 Theoretical framework

### 2.1 ADHD and inattention

In this study, the focus is specifically directed toward students with ADHD experiencing the core symptom of inattention (Tarver et al., 2014; Gray et al., 2017; Arnold et al., 2020; Smith et al., 2023). A neuropsychological understanding of *attention* frames it as capacities or processes for how an organism becomes receptive to stimuli and how it begins processing internal or external stimulation (Parasuraman, 1998). Within an educational context, *concentration* is a broader concept encompassing both ability (capacity) and effort, where *effort* refers to behavior and may comprise a person’s energy and motivation to engage (Løhre, 2021). Throughout the text, the terms *attention* or *inattention* are mainly applied in theoretical parts, while in the results section, the term concentration is used to mirror the terminology expressed by the participants during the interviews. Related to the findings, the different terms are elaborated on in the discussion.

Even though students with ADHD symptoms exhibit great diversity, a large body of research has reported deficits in neuropsychological and emotional functioning as well as in psychosocial behavior and interactions with peers (Tarver et al., 2014; Champ et al., 2023). Many of the symptoms lead to great challenges with planning, initiating tasks, maintaining focus, and managing transitions between activities, all skills that are central to self-regulated learning. As Reddy et al. (2018) point out, when such challenges inhibit the regulatory process, they potentially lead to frustration, disengagement, or reduced self-efficacy. Students with ADHD benefit from teaching approaches that enhance their skills in these areas, especially due to the emphasis on behavioral, metacognitive, and affective aspects of SRL (Reddy et al., 2018).

With respect to motivation, students with ADHD symptoms are found to be less motivated than their peers, showing less persistence in activities, preferring easier work, relying more on external standards, and, in general, seeming to find less enjoyment in learning (Carlson et al., 2002; Morsink et al., 2021; Smith et al., 2023). Furthermore, it is observed that students with ADHD symptoms display less on-task behavior than most classroom peers (Kofler et al., 2008), having shorter attention spans and seeming to be less engaged in classroom activities overall (Fleming et al., 2017; Rogers and Tannock, 2018; Arnold et al., 2020). However, when it comes to self-perceptions of competence, the results differ. Some studies find no group differences between students with ADHD symptoms and typically developing peers (Eisenberg and Schneider, 2007), whereas others report substantial group differences in perceived competence (Rogers and Tannock, 2018).

A qualitative study of students with ADHD symptoms demonstrated great individual differences in motivation, both between the students and related to different topics for each student (Løhre et al., 2021). There was, however, a clear pattern in motivation. When the tasks or topics were perceived as enjoyable, the motivation and concentration were high; with less perceived enjoyment, the motivation and concentration were correspondingly lower. The study suggests that aspects of play and enjoyment might be especially important to students with ADHD symptoms. This suggestion is underscored by Champ et al. (2023) who claim that “understanding interest and its role as a motivational factor in ADHD is key to gaining a new perspective on ADHD behavior” (p. 586).

To reach an alternative understanding of neural processing and other characteristics related to ADHD, Champ et al. (2023) propose applying a framework grounded in SDT. Rogers and Tannock (2018) used the SDT framework when they explored autonomy, competence, and relatedness in groups with high and low ADHD symptomatology, denoted *ADHD group* and *non-ADHD group*, respectively. Compared to the non-ADHD group, students in the ADHD group perceived less autonomy support, less competence, and less relatedness. Furthermore, Rogers and Tannock (2018, p.1357) find greater individual variations in perceived autonomy support and competence among students in the ADHD group than among the non-ADHD students. Based on (i) a presentation of SDT as a comprehensive motivational framework and (ii) a description of current motivation-related ADHD theories and research, Morsink et al. (2021) suggest that the role of internal motives and the relevance of SDT for students with ADHD symptoms must be explored further through intervention studies.

### 2.2 Student autonomy, intrinsic motivation, and autonomous self-regulation

In SDT, human behavior is conceptualized as internally motivated (intrinsic motivation), reflecting an “inherent tendency to seek out novelty and challenges, to extend and exercise one’s capacities, to explore, and to learn” (Ryan and Deci, 2000b, p. 70). Furthermore, SDT proposes that intrinsic motivation arises when the social environment provides support for the satisfaction of three basic psychological needs: autonomy, relatedness, and competence (Deci and Ryan, 2000; Ryan and Deci, 2017, 2020). The need for competence involves the experience of mastery while completing a learning task, while the need for relatedness concerns feeling connected to peers and teachers. Autonomy is deemed of utmost importance, as it connotes an inner endorsement of one’s actions, as a sense that they emanate from oneself and are one’s own will (Deci and Ryan, 1987, p. 1025; Ryan and Deci, 2020). Autonomous functioning entails the freedom to choose actions aligned with one’s interests, values, and sense of meaning arising from internal self-concept (Ryan and Deci, 2017). At a phenomenological level, human autonomy manifests as “the experience of integrity, volition, and vitality that accompanies self-regulated action” (Deci and Ryan, 2000, p. 254). Based on this foundation, the concept of *autonomous self-regulation* is introduced, juxtaposed with behavior that is externally controlled, either by coercion or social conviction (Deci and Ryan, 1995). At a fundamental level, autonomous self-regulation essentially revolves around expressing one’s “true self” (Deci and Ryan, 1995, p. 35). *Externally motivated behavior* refers to doing something because it leads to a separable outcome (Ryan and Deci, 2000a); for example, students might do something because they fear a teacher’s sanction or because it leads to something they find valuable, such as a chosen career. Autonomy is thus present even in externally motivated behavior, but contingent on the extent to which the value of the activity has been internalized, conceptualized as *autonomous extrinsic motivation* (Ryan and Deci, 2020a, p.62). This is a universal tendency wherein individuals assimilate the value and significance of the behavior prevalent within the social context (Ryan and Deci, 2000a).

In a school context, an autonomy-supportive teacher facilitates students’ autonomous regulation by allowing them to feel competent, related, and autonomous, thereby satisfying their psychological needs (Ryan and Deci, 2000b; Reeve and Cheon, 2021). Moreover, autonomy support promotes a sense of choice, volition, and freedom from excessive external pressure (Ryan and Deci, 2000b, p.74). As such, autonomy support possesses an important relational dimension, where teachers engage with students and (1) adopt their perspective, (2) welcome their thoughts, feelings, and behaviors, and (3) support their capacity for autonomous self-regulation (Reeve, 2009). A clear structure in the learning environment is a crucial component of autonomy support, ensuring that students are well-acquainted with the expectations and requirements placed upon them (Jang et al., 2010).

Based on SDT, researchers have investigated the synergistic relationship of students’ perceived teacher autonomy support and the provision of structure in the prediction of self-regulated learning (Sierens et al., 2009). SRL is seen as a key to student school success and involves goal-directed activities in three interdependent sequential phases: forethought, performance control, and self-reflection (Zimmerman, 2000), any or all of which can either facilitate or inhibit the regulatory process. In line with SDT (Deci and Ryan, 2000), structure needs to be combined with a certain amount of autonomy support in order to have a positive relationship with SRL (Sierens et al., 2009). Furthermore, SRL models are considered conceptually significant for students due to their engagement in self-reflection according to their own (meta)cognitive, motivational, and affective strategies used to attain the desired learning outcomes (Zimmerman, 2000; Panadero, 2017).

According to the concept of structure in the SDT tradition, SRL can be implemented in a learning environment structured around the three phases of forethought, performance control, and self-reflection, with the expectations that come with each clearly communicated to the students. We will return to this when presenting the intervention upon which our study is based.

## 3 The study context

In line with recommendations for research in this field, we provide details about the context of the study and the intervention used (Graham, 2017).

This study was conducted in a primary public school in Norway. In Norway, inclusive education is prioritized, and legislation supports the right of all students to participate and learn in regular classrooms in local schools (Nes et al., 2018). This means that teachers in Norwegian schools face a very diverse group of students in their daily work, something which places a significant demand on their teaching competence. To ensure that students with diverse needs and abilities experience inclusion, *tilpasset opplæring* (adapted education) is a fundamental principle outlined in the Norwegian Education Act (Norwegian Ministry of Education and Research, 2024) with the goal of promoting inclusivity through variations and adjustments to the diverse prerequisites and needs of the students. Importantly, this principle does not advocate for pure differentiation according to the individual student but emphasizes striking a balance between individualization and promoting each student’s sense of belonging in the community (Norwegian Directorate for Education and Training, 2024).

The study took place within the framework of a one-year intervention in which teachers were instructed by the first author to implement a didactic model for adapted education (the TIL Model) one day each week (TIL Day). TIL, then, is an abbreviation of the Norwegian term *tilpasset opplæring* (Skaalvik and Skaalvik, 2021, p.248) and translates as “adapted education.” One of the intentions of the model is developing autonomy supportive teaching practices. In accordance with SDT, teacher autonomy support promotes students’ sense of choice, volition, and freedom from excessive external pressure (Ryan and Deci, 2000b), but only if the learning environment has a clear structure so that the students are well-acquainted with the expectations and requirements placed upon them (Jang et al., 2010). In the TIL Model, structure in the learning environment is facilitated through a working plan consisting of three interdependent, sequential phases: forethought, performance control, and self-reflection (Zimmerman, 2000, p.16).

The TIL Model consists of a comprehensive work plan prepared by the teachers that provides the students with an overview of the TIL Day’s sessions (time and place), subjects, assignments, learning activities, individual work, student collaboration, teaching sessions, and common classroom activities. TIL Day activities are intended to be diverse, spanning subjects and with varied cognitive workload. As such, some activities are more practical, some more creative, some collaborative, and others requiring independent work, but all with the intention of catering to the needs of individual students. To accommodate the students’ different prerequisites and needs according to workload on the TIL Day, tasks and activities are divided into “first priority” (mandatory) and “second priority” (voluntary), but the plan also allows students to take breaks whenever they need wish. Furthermore, the plan is structured so students perform learning activities throughout the day according to the three phases of self-regulated learning (SRL) as expressed by Zimmerman: planning, working, and evaluation (Zimmerman, 2000, p. 16).

While the TIL Plan provides a clear structure, giving students an overview of the day and what is expected of them according to the three phases of SRL, it also offers them a high degree of flexibility meant to encourage them to choose and act autonomously, according to their own prerequisites and needs. This involves setting both personal and common goals with peers, establishing timelines, setting priorities for the different tasks, activities, and effort, executing tasks both alone and together with peers, and taking breaks when needed. Ultimately, the students are asked to reflect on their experiences throughout the day, focusing on the choices they made, whether those choices worked well or not, and also on how they perceived their workload and academic level. This aligns with Skaalvik and Skaalvik (2021), which aims to support students in developing a positive attribution pattern by enabling them to experience that they, with support from their teachers, are capable of influencing success and failure on TIL Days.

## 4 Method

This study was part of the larger TIL intervention involving multiple schools, teachers, and students. Of the 115 students who participated in the intervention, 60 were invited to share their experiences through interviews. Those invited were recruited through their teachers based on “student diversity” as a criterion. The students were informed that participation was voluntary and that they could withdraw at any time and without any consequences. Among the 40 students who agreed to participate, only two had a formal ADHD diagnosis. These two students and their parents, as well as their teachers, all signed an informed consent agreement. The study was registered at the Norwegian Agency for Shared Services in Education and Research (Sikt) under the number 435104.

Our choice of participants in the context of TIL Model intervention responds to Graham’s (2017) recommendations regarding direct involvement of students with ADHD diagnoses in research, and to his call for exploring better ways to translate our knowledge into practice. In accordance with recommendations by Graham (2017), we share descriptions of the study participants.

The students and their teachers were from the same urban primary school, where approximately 500 students are enrolled. The first student was 10-year-old Benjamin and the second is 11-year-old Sophie (pseudonyms). Both students have been diagnosed with ADHD in accordance with the national professional guidelines (Norwegian Directorate of Health, 2022) through the Child and Adolescent Psychiatry specialist health services. According to both the students and their teachers, the ADHD symptom of inattention is the main challenge both students face when learning in school. Even though students in Norwegian schools encounter several teachers daily, each student has a teacher with specific responsibility for following up with that student (their “contact teacher”). The contact teachers were recruited for the study when their student’s participation was secured. Both teachers have a four-year general teacher education. Sophie’s teacher (age apprx. 30 years) is relatively newly qualified and has been Sophie’s contact teacher for 2 years. Benjamin’s teacher (age apprx. 40 years) has been teaching for nearly 13 years and Benjamin’s contact teacher for 3 years. Both Sophie and Benjamin’s classes consist of about 20 students.

To ensure fidelity of the intervention (Swanson et al., 2013), the teachers involved in the study received guidance on the TIL Model and the content of the working plan and how both were intended to address adapted education in terms of differentiation and students’ participation in the community. Several pedagogical principles intended to ensure adapted education were highlighted, such as the principles of differentiation in terms of academic level, practical and creative tasks, self-selected breaks, and the balance between individual work, working with a study buddy, and group collaboration. The teachers were also instructed in autonomy-support, structure and the three phases of SRL in line with the intention of the TIL Model (Skaalvik and Skaalvik, 2021). The teachers and the one of the researchers met on three occasions during the year to reflect on how each teacher experienced student autonomy with respect to the teachers’ own practices in the framework of the model and autonomy support. Before each meeting the researcher observed in the classroom throughout TIL Day, so that joint reflections were connected to their real experiences from that day.

To support an inductive approach, two different interview guides consisting of open-ended questions were prepared: one for the students and one for the teachers.

The student interview guide explored how students experience autonomy in learning activities throughout TIL Days, with a particular focus on volitional choice, motivation, and self-regulation. Key topics included:

- General school experience and social relationships
- Perceptions of TIL Day: enjoyment, challenges, and preferences
- Planning and decision-making: choosing task order, initiating work, and managing time
- Motivation and effort: reactions to success and failure, perceived mastery
- Concentration and distractions: internal and external factors
- Interaction with peers and teachers: support, collaboration, and feedback
- Self-assessment and reflection: use of evaluation forms and perceived effort

The teacher interview guide focused on teachers’ experiences with the TIL Model and their observations of students with ADHD, particularly regarding autonomy support, motivation, and self-regulated learning. Topics included:

- Teacher background and classroom context
- Implementation of the TIL Model: opportunities and challenges
- Perceptions of the focus student: positive experiences, challenges, and engagement
- Students functioning on TIL Days vs. regular days: attention, social interaction, and emotional responses
- Observations of self-regulated learning: planning, task execution, and self-assessment
- Support strategies: task differentiation, collaboration, and teacher-student interaction
- Reflections on how the TIL Model supports or challenges students with ADHD

Each interview took place at the school, during school hours, and lasted between 45 and 75 min. Prior to the interviews, both students and teachers were reminded that their participation was voluntary and that they had the option to withdraw at any time without any consequences. All interviews were audio recorded and transcribed by a research assistant. In order to treat the students as experts on their own thoughts and reflections, we decided to read the transcripts of student interviews first, followed by those of the teachers, throughout the process of analysis. The aim of the intervention was to empower the children participating and contribute to positive changes for them. However, in line with Robinson and Taylor (2013), we were mindful of the issue of unequal power dynamics between the researchers and teachers as adults, and the two students as children. This became a concern during the analysis when questions arose regarding whether autonomy in learning activities might result in more drawbacks than benefits for these students. We address this crucial issue in the discussion (Autonomy in learning activities: A double-edged sword) as well as in the concluding remarks (regarding decisive teacher competence).

## 5 Analysis

Given the study’s exploratory nature, our aim was not generalizability or theoretical contribution, but in-depth insight into the research question. While full data saturation was not feasible, thematic redundancy across interviews suggests sufficient depth for the study’s purpose (Tjora, 2019). The analysis process was followed by collaborative discussions during each step, to compare, refine, and validate codes, code groups, and main topics. This iterative approach ensured analytical consistency and strengthened the credibility of the findings (Tjora, 2019).

In accordance with the open research question and inquiry approach, the initial data analysis was open or inductive in nature (Tjora, 2019). While reading through the transcriptions individually, we noted our spontaneous reflections about *the essence* of the participants’ stories. The notes reflect the students’ appreciation of “deciding for themselves” on TIL Day, as well as their experiences of challenges related to inattention.

In the next phase, we identified words and expressions indicating meaning and labeled them with empirically close codes (numbered) according to both the students’ appreciations and the challenges (Tjora, 2019, p. 29). After conducting coding tests which included multiple readings of the transcriptions, further empirically close coding, and adjustments to codes, we discerned several codes that had internal thematic connection to different preliminary “code groups” (Tjora, 2019, p. 37). When we summarized the various preliminary code groups at one point, we discovered that they were related to two seemingly contradictory main topics according to “students’ own will.” On the one hand, both students and their teachers expressed that they, the students, were *prevented from acting* on the basis of their own will, when facing the experience of losing concentration (challenges). On the other hand, they talked about experiences of *taking action* when relating to positive experiences of autonomy and teachers’ autonomy support in learning activities on TIL Days (opportunities). Therefore, at this point, the main topics were labeled as preliminary main topics (1) Challenges: Experiences of concentration as being beyond one’s own will and (2) Opportunities: Motivation to act on the basis of one’s own will. In subsequent analyses, we moved back and forth between the empirical data, theory, and previous research (Tjora, 2019, p. 39), and realized that these two main topics provided new insights in line with the research question.

Although the two preliminary main topics appeared to be valid at this point, it remained unclear how they reflected the students’ experiences *throughout* the TIL Day. Consequently, in line with the analytical distinction between challenges and opportunities, we again turned to the codes and code groups and performed adjustments. Finally, using Tjora’s (2019), p. 38 terminology, we unified meaningful and still empirically close main topics with underlying code groups according to the students’ experiences of autonomy in learning activities: (1) Challenges with concentration, choices, and carrying things out, (a) *Losing concentration*, (b) *Becoming disturbed and uncertain*, and (c) *Struggling to stay on track*, and (2) Opportunities of motivation and self-reflection, (a) *The freedom to choose and act*, (b) *The powerful feeling of mastery*, and (c) *Teacher support and ongoing self-reflections*.

Table 1 provides insight into the analysis process according to main topic 1 and code group 1.a.

**Table 1:** Development of main topic 1 and code group 1.a.

Spontaneous reflection notes	Empirically close coding	Preliminary grouping of codes	Preliminary main topic	Final main topic and code group
Challenges related to inattention and autonomy in learning activities	**___** Students 1 I lose concentration by myself. 2 Wandering thoughts, messing around 3 Falling out 4 I’m sort of in my own world. 5 I’m losing focus. 6 I lose concentration. It just happens. 7 I cannot focus on what to do. Teachers 8 Thoughts are everywhere. 9 Zero concentration 10 It’s not about abilities, it just happens. 11 Even though he finds it exciting, he loses focus.	**___** 1, 3, 5, 6, 9, 10, 11 Concentration beyond own will 3, 7, 10, 11 Refrained from acting based on volition 4, 7, 9, 10, 11 No solution 2, 8 Left to chaos of thoughts	(1) Challenges: Experiences of concentration as being beyond one’s own will **___**	(1) Challenges with concentration [choices and carrying things out] ___ Losing concentration

## 6 Results and interpretations

### 6.1 Challenges with concentration, choices, and carrying things out

#### 6.1.1 Losing concentration

Both students were able to describe their challenges with maintaining concentration. Sophie provides detailed descriptions of being unable to concentrate:

I lose concentration by myself; it’s like my thoughts wander around in my head and they go to the wrong place. Then they start messing around in the wrong place where I’m supposed to concentrate. For instance, here (points to the side of her head). […] When I’ve lost concentration, I’m sort of in my own world.

Here, Sophie describes losing concentration as if it is an internal process beyond her ability to control, like the mind wandering (Becker and Barkley, 2021). She details the experience as if it arises because she cannot override it, as her thoughts spontaneously start disrupting her, making it difficult for her to maintain concentration on the task at hand. By pointing to a specific area on the side of her head, she possibly indicates a localized sensation that hinders her concentration.

Benjamin expresses that he is not able to think about what he should do, according to the learning activities on TIL Days. His teacher elaborates on this:

His thoughts are a bit everywhere […]. He has zero concentration. […]. It’s not that his abilities are lacking; it just happens.

The teacher’s choice of phrasing “thoughts a bit everywhere,” “zero concentration,” and “just happens,” gives the impression that concentration is beyond Benjamin’s will, or *volition*, which is the term used in SDT (Deci and Ryan, 1995, p. 37). When his teacher points out that this happens despite his abilities, it brings to mind Parasuraman’s neuropsychological or organismic understanding of inattention viewed as a non-volitional deficit in the ability to receive and process internal or external stimuli (1998).

#### 6.1.2 Becoming disturbed and uncertain

Both Sophie and Benjamin describe how their struggles with concentration make them easily distracted and uncertain according to volitional choice in learning activities, which in turn hinders their ability to plan and get started on TIL Days:

Sophie: Making choices is difficult for me. […] To decide which tasks to start with is challenging, because I become uncertain of what is the best choice. […] It is also challenging to get started. Sometimes it’s OK that I can make some decisions on my own, but I cannot consistently manage that.

Benjamin: Planning and getting started are both challenging. […] Occasionally, other students disturb me… or I think I might be the one disturbing them […] Sometimes I get distracted when my friends laugh loudly or do something funny. Then I look at them, and I become slightly curious.

Here, Sophie is concerned about uncertainty in making volitional choices and decisions because she is unsure about what the best choices are for her. Her statement that she finds it OK to make decisions on her own some days but not others, highlights how students with inattention may function differently within and between days (Kofler et al., 2008). Benjamin, for his part, links distractions as following from interactions with peers. However, both students expressed difficulties with “planning and getting started,” as Benjamin puts it—something that may be understood in light of their uncertainty related to making volitional choices at the beginning of the TIL Day.

Although Sophie’s self-reflections comprise negative emotions, she simultaneously expresses valuing interest and enjoyment (Løhre et al., 2021; Champ et al., 2023) when she considers what suits her best:

I can choose those tasks I am confident about first, and those I enjoy the most, which makes things a bit easier. […] If I do, it feels like a break! […] I can also decide to begin with the worst tasks first to get them out of the way, but if I do the math before eating time, I often lose concentration and then I want food and to do other things, and then I become very like this (puts her head down on the table) because I get really tired.

Sophie’s reflections highlight how uncertainty related to making autonomous choices stems both from her struggles in math and from her attention and concentration difficulties. When she reflects on what is best for her—whether to choose the tasks she finds enjoyable and interesting first, or the ones she finds difficult—she points out that the difficult ones might have an advantage, since it could feel good to have them “out of the way.” At the same time, she seems very aware that if she chooses to do math first—the subject she struggles the most with—she may risk losing concentration, running out of energy, and ending up struggling throughout the day. Even though this could naturally apply to many students who find math challenging, Sophie appears to be particularly conscious of the fact that doing math tasks requires so much thinking effort (cf. Champ et al., 2023, Figure 10, p. 588), that she is actually better off choosing the opposite—“those [tasks] I enjoy the most.” In doing so, she imagines she can have a good time for a while, almost “a break,” in her own words.

#### 6.1.3 Struggling to stay on track

The two students also relate loss of concentration to how they experience the qualities of the learning activities. In line with previous research (Løhre et al., 2021; Champ et al., 2023), both Sophie and Benjamin find it easier to concentrate and expend less effort to do so when they find the tasks interesting or enjoy the activities. When the tasks give little joy or are challenging, they tend to lose energy and concentration. Sophie explains:

At times it’s a bit difficult to engage in something I do not find very enjoyable because I tend to lose concentration. I’m not very fond of math. I do not consider myself to be very skilled in math and I do not find it enjoyable. That’s because I must put quite a bit of effort into thinking and sometimes, I lose my concentration.

Here, Sophie describes experiencing a loss of concentration if she does not find the task enjoyable or if the task requires a lot of cognitive effort. Furthermore, it’s interesting how Sophie explains that math is not enjoyable since she considers herself to have poor skills in this subject and thus it requires too much cognitive effort. Even though task/activity enjoyment may be present initially, this can be lost if too much cognitive effort is required and the feeling of competence is lacking. Conversely, Benjamin’s teacher describes his loss of concentration as occurring regardless of whether the tasks are cognitively demanding or not:

His schoolwork suffers because he cannot maintain concentration on what he’s doing. Even if he finds the tasks interesting, he struggles to pay attention. He loses focus and starts thinking about his own things and then gets eager to talk about them in class.

The teacher’s statement that even interesting tasks do not hold Benjamin’s attention echoes previous research showing that these groups of students have short attention spans and behavior reflecting low engagement (cf. Rogers and Tannock, 2018). Variations in these students’ engagement related to their interest and joy in tasks, on one hand, and the degree of cognitive effort needed, on the other, is well known in the field (cf. Champ et al., 2023). Despite reacting differently vis-à-vis engagement and cognitive effort, both students in this study have in common that they fall off track and easily slide into off-task activities (cf. Kofler et al., 2008). The teachers’ perceptions support the students’ own experiences:

Sophie’s teacher: The increased flexibility in structure and the reduced teacher-driven nature of learning activities on TIL Days pose challenges for Sophie in maintaining concentration and staying on track. Her self-selected breaks sometimes become excessively prolonged, with statements like “Oh, I lost track of time…” Consequently, prioritizing tasks for completion becomes difficult, leading to a lack of mastery experiences.

Benjamin’s Teacher: While other students focus their energy on completing tasks, he expends his energy on staying focused on the fact that he indeed has tasks to complete and what they entail, rather than actually completing them. […] He often expresses feeling incapable of handling it […] and his main challenge lies in maintaining self-driven motivation throughout the day.

According to Sophie’s teacher, TIL Day offers a more flexible learning environment with less teacher control. This aligns with the TIL Model’s intention for students to exercise free choice and actions, and for teachers to practice autonomy support (Skaalvik and Skaalvik, 2021). The problem, from the teacher’s perspective, is that less external control for Sophie leads to a loss of concentration and challenges in “staying on track” or *being self-regulated*, which is the term used in the ADHD field (Champ et al., 2023). When her teacher adds that Sophie loses track of time during self-chosen breaks, this aligns with other research showing that ADHD students under-estimate the passage of time (Arnold et al., 2020). When it comes to Benjamin, his teacher’s statement suggests that increased freedom challenges him as well, but in a different way. When Benjamin expends all his energy orienting himself within the TIL Plan and figuring out what tasks to do and how to do them, he does not have energy left to actually engage in and complete tasks. Thus, it is not necessarily just self-regulation that becomes difficult for him due to the freedom on TIL Day, but also *self-regulated learning*. In addition, Benjamin’s statement highlights that even when he, at some point, knows what he is supposed to do, he lacks the initiative to actually do it, a well-known challenge for students with inattention (Champ et al., 2023). Again, this is illustrative of ADHD-specific impairments among students in school.

According to Reddy et al. (2018), the frameworks for self-regulation and self-regulated learning for students with ADHD overlap in that both are typically viewed as goal-directed activities and involve behavioral, affective, and metacognitive subprocesses that can facilitate or inhibit the regulatory process. Since all these subprocesses seem to challenge Sophie and Benjamin, the students are at risk of not having mastery experiences in the regulatory process, which in turn can lead to negative self-attribution. Consequently, they may struggle to initiate learning activities and maintain self-driven or intrinsic motivation throughout the day, as Benjamin’s teacher points out. A question that arises at this point is whether the “added” expectation to be self-regulated *in learning* due to autonomy challenges them to such a degree that a more traditionally fixed environment and teacher control might serve them better.

### 6.2 Opportunities in terms of motivation, support and self-reflections

#### 6.2.1 The freedom to choose and act

Despite facing challenges throughout TIL Days, Benjamin and Sophie still express appreciation for the opportunity to make volitional choices in learning activities:

Benjamin: It’s a little more fun on TIL Days because I can decide a bit more for myself […] I prefer these days over other days because I can choose which subjects to work on. It’s cool that we get to decide when we have our break, and sometimes we do not even have to go outside… and sometimes, for example, I can go out just with a friend. I like doing things a bit differently than on regular days.

Sophie: Finally, I get to decide for myself, then I’m happy! […] I like TIL Days because I can decide what is the best for me, such as which tasks I prefer to do first and so on. For example, I can choose whether I want to do the most difficult tasks or the easiest tasks first. I want to choose what to do myself and it’s nice being able to.

Given the challenges these students face on TIL Day, their appreciation of “deciding for themselves” could be interpreted in different ways—either as a genuine sense of novelty and excitement compared to more structured school days, or as a response shaped by the interview context, possibly reflecting what they believed the researcher wanted to hear. At the same time, their choice of words evokes associations with autonomy as a fundamental psychological need, connoting an inner endorsement of one’s actions and as sense volition (Deci and Ryan, 1987, p. 1025). Even if the students’ use of words such as “favor,” “cool,” “fun,” “like,” “finally,” and “happy” appears paradoxical, given the challenges following from autonomy, as described previously, their statements suggest the potential of autonomy in learning activities in promoting wellness, which is in line with Champ et al. (2021, 2023). While Benjamin highlights the value of making his own decisions and having the freedom to choose *per se*, Sophie points out that this gives her the opportunity to cater to her own preferences and needs.

Benjamin’s teacher elaborates on why Benjamin appreciates the freedom to choose:

He is very happy that he is allowed the freedom to choose. It suits him well. […] He likes being able to choose the order of the tasks and what he wants to do. […] Another advantage for him on TIL-Day is that he does not have to sit still and listen to a teacher. He appreciates it because then he does not have to feel the straitjacket of the traditional school system. […] [He’s] not being pressured into that typical, rigid school situation.

Benjamin’s teacher believes, in agreement with Benjamin, that it is the freedom to choose the order of his tasks and what to do that makes him happy on TIL Days. Furthermore, she points out that TIL Days contribute to him being more active in the learning activities as compared to regular days which have a more traditionally rigid classroom structure, where the teacher is active and Benjamin has to sit still and listen. Accordingly, she notes that the flexibility of TIL Days allows Benjamin to avoid feeling constrained and pressured.

When it comes to Sophie, her teacher points out that Sophie benefits from deciding for herself because it motivates her to exert additional effort:

What motivates Sophie the most on TIL Days is that she gets to make her own decisions like “Now I get to choose.” […] She gets an extra gear or a killer instinct because she is the skipper who controls the ship. It’s her day, in a sense. She can initially feel a bit demotivated when receiving the TIL Plan, unsure if the tasks align with her preferences. However, compared to regular school days, she seems to think, “Alright, this is sort of my day!” which quickly transitions into a positive outlook.

According to Sophie’s teacher, autonomy in learning activities results in a sense of agency and “ownership” of the day for Sophie, which is exactly the word Ryan and Deci use with respect to autonomy: “a sense of initiative and ownership in one’s actions” (Ryan and Deci, 2020, p. 1). Furthermore the teacher points to the connection between Sophie’s sense of ownership and her increased motivation on TIL Days, which also resonates with SDT (Deci and Ryan, 1987).

While Sophie experienced uncertainty regarding what benefited her most during the first phase of the intervention, her teacher observes that this has changed throughout the year:

She knows herself quite well now, often making good choices and structuring the day in a manner that suits her. She frequently chooses to do the tasks she’s confident about first, just to get started. [.] Then she saves the more challenging tasks for the last session. Consequently, she enjoys TIL Days and sustains motivation, even if the math can be challenging toward the day’s end.

Here, Sophie’s teacher demonstrates trust in and support for Sophie’s strategy of postponing difficult tasks, acknowledging that Sophie knows herself best in terms of how to get started and sustain motivation throughout TIL Day. Moreover, she explains that Sophie knows best when it comes to the need for breaks as well:

She becomes more motivated when she can take breaks without feeling observed or facing teacher directives such as, “Now is not the right time, so you’ll need to wait.” It’s all about timing and concentration. So, when she senses tiredness, she can have a break. Consequently, she’s more motivated to complete tasks on the plan, even if they aren’t her favorites.

Since all students have the freedom to make choices in learning activities on TIL Days, Sophie is relieved from the pressure of adhering to conventional rules regarding work time and breaks. Seen from her teacher’s perspective, this presents a new opportunity for her to align her energy levels with her concentration and need for breaks. While Sophie may perceive her concentration as something beyond her own control, the newfound flexibility on TIL Days can nevertheless potentially give her a sense of carrying out actions based on her own will. This, in turn, could explain her motivation, even when dealing with challenging tasks, as noted by her teacher. According to SDT, autonomous actions are described similarly, as “an inner endorsement of one’s actions, a sense that they are emanating from oneself” (Deci and Ryan, 1987, p. 1033), and thus the opposite of being externally determined, controlled, or “directed,” to use the teacher’s word.

When asked how she copes with losing concentration on TIL Days, Sophie elaborates on how the solution lies in her motivation to complete tasks:

At times, managing it on my own can be quite challenging. However, I can think along these lines: “Alright, it’s time to concentrate. Which subject do you want to start with? Which one do you prefer?” […] So, I think like this: “I’m going to complete this task, then I can be proud of myself, and then it’s going to be a great day.” […] It’s quite motivating. I tell myself that I must work hard, avoid distractions, and really concentrate on the tasks. Furthermore, I think, “If I have any questions, I should ask for help.”

This highlights the strategies that Sophie has learned to maintain motivation throughout the TIL Day, transitioning from completing tasks she feels confident about during the initial phase to anticipating positive outcomes, such as feeling proud and having a good day, by the final phase. This observation aligns with SDT and its distinction between intrinsic and autonomous extrinsic motivation (Ryan and Deci, 2020). Furthermore, Sophie’s awareness that she can ask for help when needed also aligns with SDT’s assertion that autonomy does not mean being independent, but rather being “autonomously dependent” (Ryan and Deci, 2006, p. 1562).

#### 6.2.2 The powerful feeling of mastery

Given that both Sophie and Benjamin are at risk of not having mastery experiences in self-regulated learning during TIL Days, potentially threatening their self-worth, experiences that provide them with a feeling of mastery may prove to be all the more significant. When it comes to Benjamin, his teacher confirms this:

There has been a lot of variation in his experiences of mastery. He becomes extremely happy and proud when he does master something. […] When he masters and achieves what I think he should be able to do… he is extremely satisfied. Also, if he gets praise for something he has done, which is good by his standards, he is super happy, and he is extremely proud if he completes all the first priority tasks on TIL Days.

The teacher’s use of superlative adjectives when describing what mastery experiences mean to Benjamin serves as a reminder that achieving such experiences does not come easily to him *on TIL Days*, given his ADHD diagnosis and the challenges he faces with concentration.

The feeling of mastery, or the sense that one can succeed and grow, is important for intrinsic motivation in learning, according to SDT (Ryan and Deci, 2017, p. 11). Benjamin describes this in his own way:

Sometimes I just believe in myself and then I do it. Usually I think, “I cannot do it,” but if I think “I can,” then I do! […] It’s like when you manage to do it, you find it fun. When you cannot do it, it’s boring […] When I complete a task, I find it fun and then it becomes even more enjoyable.

Here, Benjamin seems to focus on his own experiences of the “power of thought.” Given his previous experiences of not mastering learning activities, he may have developed a meta-awareness in that he often thinks “I cannot” or “I will not be able to do it.” When he then experiences mastery, he is given the opportunity to be aware of new thoughts coming up, such as “I can” or “I did it.” In this way, he might be aware that his thoughts, either positive or negative, significantly impact what he actually achieves, something which is well confirmed through research in the field of motivation through self-efficacy (Bandura, 1997, p. 3). Furthermore, it’s interesting how Benjamin reflects on joy when mastering tasks; while he finds tasks more enjoyable when he manages to complete them, he also seems to experience a newfound joy—which is the experience that working on tasks can be fun in itself.

#### 6.2.3 Teacher support and ongoing self-reflections

Both teachers state that their students benefit from having tasks differentiated to their skill levels and needs on TIL Days, in line with the principle of adapted education on which the TIL Model is based (Skaalvik and Skaalvik, 2021):

Sophie’s Teacher: If the tasks are practical, involve physical activity, or group collaboration, the subject itself does not matter as much. It’s more about the topic within the subject and the working method that are important. However, she has slightly lower motivation in theoretical subjects because the tasks then often involve more standardized working methods.

Benjamin’s Teacher: If he gets tasks that are adapted to his skill level, whether he can manage them alone or with a bit of support, then he has a very good day. […] And if it’s a task that interests him, he can actually work in quite a focused way.

These statements highlight that differentiation of learning activities is not just about the academic difficulty level and the number of tasks and workload. It also involves ensuring that the tasks resonate with students across a spectrum of theoretical and practical content, whether they involve individual work or collaboration, and the balance between cognitive effort and physical activity. And as Benjamin’s teacher describes, working on tasks that interest him improves his focus.

In line with the students struggling to plan, get started, and stay on track on TIL Days, both teachers also highlight the students’ need for support in terms of *structure, planning, making choices*, and *navigation*:

Benjamin’s Teacher: He really cannot navigate this day successfully without my support. […] If he has set up a plan, I need to check in with him and ask, “What is your plan for today?” Often he needs a bit of help to plan and get started. […] The transitions from one task to another are the most challenging for him because they require a lot of structure.

Sophie’s Teacher: In essence, she requires substantial support in structuring her TIL Day. […] I am conscious of placing her with a peer who is better at structure, so they can create a plan and stick to it together. It’s also crucial that it’s someone she feels comfortable enough with to engage in academic discussions or to ask for help or guidance.

Since TIL Days have a more flexible structure than regular school days, greater demands are placed on students to determine their own structure. As Benjamin’s teacher describes, structuring or regulating himself is challenging for Benjamin, so she supports him through all phases of the day. The same is noted by Sophie’s teacher, but she also highlights providing support for structure through partnering Sophie with peers who handle this more effectively. Furthermore, she points out that the quality of the peer relationships should be safe enough for Sophie to feel she can engage in academic discussions or ask for help or guidance, in alignment with SDT on teachers providing autonomy support (Reeve, 2009). Here the teacher’s descriptions of support for structure in this study align with SDT, in that it is not controlling but autonomy supportive (Ryan and Deci, 2020, p. 4), something that points to a high degree of fidelity in the study’s intervention (Swanson et al., 2013).

Additionally, both teachers highlight that support also involves ongoing dialog with their students throughout the day, and here Sophie’s teacher explains:

We collaborate as a team to plan the day, asking questions like “What would you like to do then?” and “What do you think is a good idea to do?” Afterwards, we usually discuss it, and I point out to her, “It’s great that you solved it this way” and we talk about how it feels and what it means for her. I ask if she wants to do it this way again, if she will try to remember it for next time and ask her to carry this forward to the next TIL Day. […] One day she came up to me and said, “I think math is my Mount Everest.” So we talked a bit about what that means and then we discussed how it’s possible to climb Mount Everest, so it’s also possible to succeed in math. She was like, “Yes, maybe it is,” even though it can feel difficult or unattainable.

Sophie’s teacher describes the dialogic support she provides as “teamwork.” By asking Sophie questions, she gives her the opportunity to reflect on what constitutes good choices, how such choices make her feel, and how they help her achieve during the day. Furthermore, it is interesting how the teacher describes that, through dialog, she tries to create a connection between the good choices Sophie makes in the present and situations where she will face similar choices in the future. When Sophie compares math to climbing Mount Everest, it becomes an analogy for how their ongoing dialog and Sophie’s self-reflection reframe her perspective on the challenges she faces in math, making them feel both more tangible and achievable.

When Benjamin’s teacher is asked if there is something she finds beneficial for him on TIL Day, she also mentions dialog and the reflections they trigger for him:

For Benjamin, the benefit on TIL Day is that he and I can reflect together on how things are progressing throughout the day. When I sit down and talk with him, he can reflect and become more self-aware of his role on TIL Days and of his needs for support. This affords him the opportunity to practice planning, making wise choices, and navigating the TIL Day.

According to Benjamin’s teacher, providing autonomy support through reflective dialog provides an opportunity for Benjamin to recognize that he is facing challenges, help meet his need for assistance, and at least help him to navigate as a self-regulated learner not solely for the purpose of succeeding in school, but also in life, more generally.

## 7 Discussion

Through the perspectives of two students diagnosed with ADHD and their teachers, this study provides new insights into how ADHD students with inattention experience autonomy in learning activities, both on possible beneficial aspects of autonomy, and challenges.

### 7.1 Autonomy in learning activities: a double-edged sword

When the two students who participated in this study faced challenges in learning activities on TIL Day, they attributed them to a “loss of concentration,” as if it happened beyond their own volition, contrary to autonomous behavior as outlined in SDT as originating in one’s own will (Ryan and Deci, 2006). Since attention is pivotal for students’ cognitive effort in learning activities (Gray et al., 2017), and this is perceived as lost and impossible to override, the students’ psychological sense of being autonomous learners could be lost as well. If so, this may hinder their experience of both competency and relatedness to peers, which according to SDT, would threaten their motivation and learning (Ryan and Deci, 2017, p. 86). The question is then whether their “added” challenges following from autonomy in learning activities outweigh the opportunities. Might a traditionally fixed learning environment and teacher-driven learning activities be preferable after all? To answer this question, it is particularly important to examine how student autonomy in learning activities necessarily involves an expectation for students to be self-regulated, to a greater extent than in a traditionally fixed environment. Within the TIL Model, this is recognized in the way that the TIL Plan encourages students to plan, work, and evaluate in accordance with Zimmermann’s phases of self-regulated learning (2000). According to Zimmerman (2000, p. 16), self-regulated learning encompasses a range of abilities, in which many point to advanced cognitive abilities like task analysis (forethought), attention focusing (volitional control), and causal attribution (self-reflection). In addition, as shown by Sophie and Benjamin, the expectation of self-regulated learning challenges regulatory subprocesses, such as struggling to initiate learning activities (behavioral), experiencing uncertainty about choices and time (metacognitive), and feeling incapable (affective), which is in line with Reddy et al. (2018). Accordingly, we should also bear in mind that autonomy depends on complex neurocircuitry in the form of “integrative processing of possibilities and a matching of these sensibilities, needs and constraints” (Ryan and Deci, 2006, p. 1565). Thus, since the “added” requirement for self-regulation that comes with autonomy in learning activities might challenge all students in areas where they struggle, we suggest it could pose a serious threat to these students’ self-concept and sense of competence, in which case autonomy might tip from being a source of wellness and growth to becoming a burden, much like a double-edged sword.

### 7.2 Optimism following from volitional choice, autonomy-support, and motivation

The two students who participated in this study associated autonomous behavior with positive emotions, enjoyment, freedom, and a sense of ownership, suggesting autonomy as a basic psychological need also for them. Clearly, challenges related to autonomy in learning activities do not equate to students not needing or wanting to be autonomous. Why is it that these students appreciate the freedom to choose and decide for themselves, despite the apparent contrast with the challenges they face?

First, as outlined in SDT, the freedom to choose only fosters motivation when accompanied by adequate autonomy support and a clear structure in the learning environment (Ryan and Deci, 2000b; Jang et al., 2010). In this study, these conditions were addressed within the framework of the TIL Model (Skaalvik and Skaalvik, 2021, p. 248). Teachers were trained in autonomy-supportive practices, including differentiation of tasks and learning activities in terms of difficulty level, workload, interest, and enjoyment, facilitation of peer collaboration and peer support, and the use of a working plan that provided students with both an overview and a sense of structure throughout the school day. This implies that the students’ seemingly positive experiences with autonomy in this study did not arise spontaneously, but rather as a result of substantial support over time. Without such support, the challenges would likely have been more prominent in both the students’ and teachers’ narratives.

Secondly, the assumption of autonomy as beneficial to the two students must be understood in light of significant individual variations both in their motivation styles and the kind of support needed. For one of the students, the teacher observes that the freedom to choose the order of tasks appears to act as a catalyst for an “extra gear,” as it allows her to choose to begin with tasks that interest her and bring her joy—an experience closely aligned with the concept of intrinsic motivation (Ryan and Deci, 2000a). Furthermore, when the more difficult tasks remain for the final part of the school day, the student describes herself being motivated by thinking about what comes afterward: feeling proud and happy, and having a great day. Thus, in a surprisingly nuanced way, her wording evokes associations with a shift from being intrinsically motivated to motivating herself by focusing on the outcome separate from the learning activities, though still volitional and personally valued—what Ryan and Deci call “autonomous extrinsic motivation” (Ryan and Deci, 2000a, p. 62). The other student, however, express self-efficacy arising from mastery experiences, the feeling of competence, and finally a newfound joy when working on tasks (Ryan and Deci, 2017). While the first student appears to naturally seek out challenges and shows a willingness to learn despite difficulties—reflecting an inherent tendency (Ryan and Deci, 2000b, p. 70)—the second student seems to benefit more from having tasks adapted to his needs, which depends on ‘external’ conditions in his social environment (Ryan and Deci, 2017).

Furthermore, we find it worthwhile to elaborate on whether the two students, under certain conditions, seem to benefit from the power of choice in terms of maintaining concentration throughout TIL Days. Considering that the well-established concepts of attention and inattention are defined as organismic—and thus not will-driven (Parasuraman, 1998)—we find this optimistic turn interesting. Based on our previous qualitative findings (Løhre et al., 2021), as well as quantitative neuropsychological results (Løhre et al., 2022), together with years of clinical experience, the concept *concentration*, as suggested by Løhre (2021), captures this opportunity perspective in terms of both ability and effort. While *ability* covers what the students in this study describe as beyond their own will (cf. Parasuraman’s organismic process), *effort* refers to the energy available to apply cognitive resources and motivation in order to engage, as based on one’s own will (Løhre, 2021, p. 7). According to the two students in this study, this gives reason to ask whether the power of choice enables them to balance their effort in accordance with their available energy to apply cognitive resources, need for breaks, and ability to maintain concentration. However, once again, the results in this study highlight that such energy or will to apply cognitive resources does not arise on its own but rather emerges under certain conditions in the students’ social environment(s). Although the intention of the TIL Model is to provide a clearly structured learning environment (cf. the phases of SRL; planning, working, and an evaluation phase) which can support students’ “necessary know-how to use self-regulatory strategies” (Sierens et al., 2009), this alone does not seem to be sufficient for the two students in this study. In line with Sierens et al. (2009), it is autonomy support that might provide students with the necessary energy to effectively engage in the SRL processes. We will elaborate on this in the coming section.

### 7.3 Optimism following from self-reflection

The results of this study reveal that two students derive benefits from self-reflection and potentially increased self-awareness following from the power of choice. Our analysis shows that the students are given the opportunity to reflect on their experiences throughout TIL Day, both on their own and in dialogical interaction with a teacher.

Although this appears consistent with SDT and the relational dimension of autonomy support in terms of “welcoming the student perspective, their thoughts, feelings, and behaviors, and supporting their autonomous self-regulation” (Reeve, 2009 p. 162), the particular ongoing *dialogic*, *interactive (back-and-forth)*, and *self-reflective* dimensions deserve to be explored further in the future (Reeve et al., 2018). For students with inattention, this form of support may prove especially valuable, as these students can function differently from day to day—as observed in the two participants in this study. As with one of the students, her awareness of own functioning was not only valid, but also essential for teachers to recognize and respond to.

While such teacher support is reported in this study, it must also be seen in relation to the TIL Model as a framework. TIL Days consist of a more flexible learning environment, with the increased activity and self-regulation of students giving teachers more time for such dialogical interactions with each student. In the one student’s case, experimenting and reflecting with her teacher on different choices in accordance with the task sequences not only clarified what benefited her most, but also how her motivation changed throughout the day, from what appears to be intrinsic motivation to autonomous extrinsic motivation. Overall, this suggests that autonomy support for students with inattention should constitute an opportunity wherein they are allowed to express themselves and their own needs, something associated with SDT and the opportunity that comes with autonomous self-regulation to express one’s “true self” (Deci and Ryan, 1995, p 33). To facilitate this, ongoing student-teacher dialog about the students’ experiences seems particularly valuable (Reeve et al., 2018), with the potential result of increased self-awareness regarding both challenges and opportunities, and thereby positive self-concept, enhanced learning outcomes, and wellness.

## 8 Study limitations

The TIL Model, grounded in the fundamental pedagogical principle of adapted education, forms the basis of the intervention on which this study is built—and consequently, the basis of study findings. Given different interventions, student experiences of autonomy in learning activities would vary accordingly.

While the intervention was designed to support students’ autonomy through structured and differentiated learning activities, the fidelity of implementation may have varied across the teachers and class context. Although efforts were made to ensure consistency, such as teacher training and regular follow-up meetings, variations in how autonomy support was enacted could have influenced the students’ experiences. Future studies should consider using fidelity checklists or observational tools to systematically assess implementation quality (Farmer et al., 2023).

During the broader intervention, several students expressed that they appreciated TIL Days because they were allowed to make their own choices. This positive feedback might echo intervention features described to students ahead of the implementation and hence, it might have influenced the researchers’ attentiveness to autonomy-related benefits in the present study. The choice to apply SDT, with its emphasis on psychological wellness and intrinsic motivation, could have reinforced a bias toward interpreting autonomy as inherently beneficial. The coding process was followed by joint discussions to compare, refine, and validate the codes and categories. This iterative process ensured analytical consistency and strengthened the validity of the findings (Tjora, 2019).

The small number of participants in this study allows for an in-depth understanding of individual experiences rather than generalization to the broader ADHD population, which is highly diverse. However, the study’s suggestion that autonomy causes intrinsic and autonomous extrinsic motivation for the two students involved are not based on controls or elimination of other potential reasons. Therefore, future research with larger and more varied samples is needed to support generalization. Hopefully, this study will serve as a source of inspiration in that regard and further, provide a valuable foundation for developing inclusive educational practices.

## 9 Conclusion

In this explorative study with an innovative intervention, the results of two students’ experiences and their teachers’ interpretations are thought-provoking and provide a valuable basis for further research on positive psychological factors and well-being in the field of ADHD. Both the students and their teachers describe concentration as something that can be lost, seemingly beyond the students’ own volition. Consequently, when it comes to autonomy, the students seem to experience challenges in learning activities in terms of disturbances, uncertainty, and staying on track. However, through the voices of the participants, this study offers insight into how differentiated autonomy support is tailored to individual needs and capacities and can help students engage based on personal interest, enjoyment, or a sense of competence and thereby help them experience a renewed, intrinsically driven energy to apply cognitive resources in learning.

Given that students with ADHD symptoms exhibit greater diversity than other students (Kofler et al., 2008), their ability to exercise autonomy in learning activities largely depends on the competence of teachers in providing differentiated support for both autonomy and structure, based on each student’s individual resources, prerequisites, and needs (cf. Ryan and Deci, 2017, p. 367). Furthermore, teacher and peer availability and ongoing dialogic interactions about challenges when they arise might be crucial, echoing an SDT appreciation of autonomy, not as independence, but as “autonomously dependent” (Ryan and Deci, 2006, p. 1562). Such a community-oriented approach to student autonomy is also in line with the principle of adapted education in the Norwegian educational context, where the balance between individual support and finding support within the community is emphasized (Norwegian Directorate for Education and Training, 2024). It also aligns with an upward understanding of this group of students’ endeavors to make independent choices valuable. Rather than focusing on the students’ challenges on an individual level and implementing measures aimed at addressing them, it may prove more fruitful to explore opportunities within the social context. However, given the extent of autonomy support needed due to inattention and self-regulation in learning activities, it is, in line with Panadero (2017), pertinent to question whether teachers in schools have the necessary conditions, capacity, and competence to support this ongoing differentiated and dialogical autonomy support. If not, autonomy in learning activities might burden these students and potentially result in unequal access to learning opportunities and decreased participation in school.

## Data Availability

The datasets presented in this article are not readily due to the sensitive nature of the interview data involving vulnerable children. Access may be considered on a case-by-case basis and is subject to strict ethical and legal requirements. Interested researchers may contact the corresponding author for further information. Requests to access the datasets should be directed to marit.uthus@ntnu.no.
